# Cardiac Arrest With Spontaneous Coronary Artery Dissection in a Young Female

**DOI:** 10.7759/cureus.21697

**Published:** 2022-01-28

**Authors:** Pabitra Adhikari, Osama Elkhider, Harvey Friedman, Muhammad S Akbar, Angkawipa Trongtorsak

**Affiliations:** 1 Internal Medicine, AMITA Health Saint Francis Hospital, Evanston, USA; 2 Critical Care, AMITA Health Saint Francis Hospital, Evanston, USA; 3 Interventional Cardiology, AMITA Health Saint Francis Hospital, Evanston, USA

**Keywords:** chest pain, syncope, scad, spontaneous coronary artery dissection, cardiac arrest

## Abstract

Spontaneous coronary artery dissection (SCAD) is a rare condition that has variable clinical presentations requiring a very high index of suspicion for diagnosis. We present here a case of a young female with SCAD who initially presented with chest pain and syncope, with progression to cardiac arrest.

## Introduction

We report a case of a 39-year-old Caucasian female with spontaneous coronary artery dissection (SCAD) in the left anterior descending (LAD) extending to the left main coronary artery (LMCA). SCAD is a non-iatrogenic, non-atherosclerotic condition, in which a hematoma develops in the tunica media of the coronary artery leading to separation of its wall, with a subsequent compromise of the coronary lumen causing acute ischemic event. The mechanism of the disease remains poorly understood, but it has been suggested that the hematoma can form through either, an intimal tear with blood dissecting to the medial layer, or can develop spontaneously in the setting of ruptured microvasculature within the tunica media [1]. SCAD is more prevalent among young women, with females constituting 87%-95% of all cases and an average age of presentation of 44-53 years [2].

## Case presentation

A 39-year-old Caucasian female initially presented with chest pain and a syncopal episode. The patient did not have any diagnosed medical condition, was not on any regular medication, did not smoke, drink alcohol, or use illicit drugs. She had an uncomplicated pregnancy with normal vaginal delivery three years ago. She had unremarkable physical examination findings and had a negative cardiopulmonary work-up including electrocardiogram (EKG), and cardiac enzymes. Given her young age, low-risk factor for the acute coronary syndrome (ACS) and negative workup she was discharged home with a possible diagnosis of vasovagal syncope.

The following day, the patient developed worsening chest pain and emergency medical service (EMS) was called. Upon the arrival of EMS, the patient became unresponsive and was found to be in pulseless ventricular fibrillation (VF). Immediate cardiopulmonary resuscitation (CPR) was initiated and she arrived in the ED in VF cardiac arrest with ongoing CPR. With the continued CPR, the return of spontaneous circulation (ROSC) was achieved after a downtime of 20 min. The patient was unresponsive following ROSC. Vital signs were unstable with a blood pressure of 88/40 mmHg, pulse rate of 32 beats/min, and respiratory rate of 55 breaths/min. The patient had a faint pulse and cool peripheries. Cardiopulmonary examination revealed bilateral basal crackles. Given the acute presentation in young age, differentials were ACS, pulmonary embolism, hypertrophic cardiomyopathy, aortic dissection, and arrhythmias with or without electrolyte abnormalities.

**Figure 1:**
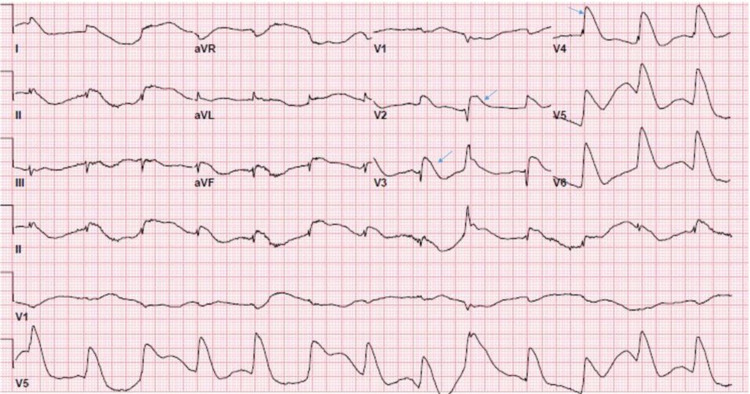
Post cardiac arrest EKG showing ST segment elevation MI in anterolateral and septal leads. EKG, electrocardiogram; MI, myocardial infarction

**Figure 2:**
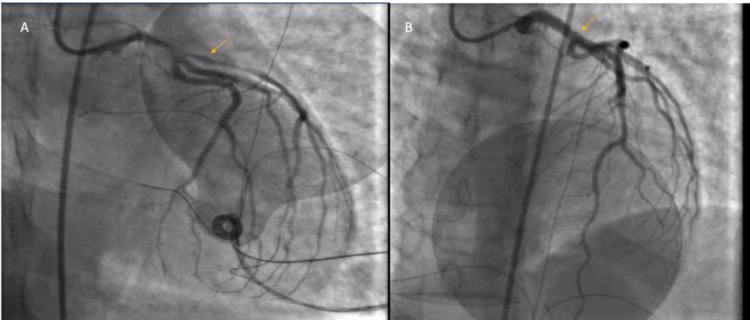
Coronary angiogram showing disruption of flow of contrast material due to SCAD of LAD artery extending to left main coronary artery (A). Coronary angiogram showing patency of flow of contrast material in LAD artery and LMCA after placement of drug eluting stents (B). SCAD, spontaneous coronary artery dissection; LAD, left anterior descending; LMCA, left main coronary artery

The EKG after ROSC revealed ST segment elevation on anterolateral and septal leads (Figure 1). High sensitivity troponin was elevated to 16,000. Renal function, liver function, and complete blood counts were within the normal limits. Toxicology screening was negative for alcohol, opioids, cocaine, cannabinoid, barbiturate, amphetamine, phenyl cyclohexyl piperidine (PCP), and benzodiazepine. Chest X-ray revealed pulmonary edema. Emergent cardiac catheterization demonstrated SCAD in LAD extending to the LMCA (Figure 2A) which required three drug-eluting stents (Figure 2B). Blood pressure maintenance required an intra-aortic balloon pump (IABP) and three vasopressors, i.e. norepinephrine, epinephrine, and vasopressin. Post-procedure echocardiogram demonstrated global hypokinesis of apex, anterior, and inferior wall with an ejection fraction (EF) of 30%. The following day she was gradually weaned off the vasopressors. Echocardiography showed an improved EF of 45%. Screening work-up for possible association with auto-immune diseases like systemic lupus erythematosus (SLE) and rheumatoid arthritis (RA) were negative. She was discharged on aspirin, atorvastatin, ticagrelor, and metoprolol including cardiac rehabilitation.

On follow-up at regular intervals, the patient developed mild chest pain after 43 days of percutaneous coronary intervention (PCI), repeat catheterization demonstrated 100% thrombosis at the site of the stent and it was managed with serial balloon angioplasty and intracoronary tirofiban. Ticagrelor was switched to prasugrel whereas aspirin, atorvastatin, and metoprolol were continued. The patient has been following up at regular intervals following the second catheterization and has been involved in cardiac rehabilitation therapy.

## Discussion

Spontaneous coronary artery dissection has been found to be associated with fibromuscular dysplasia (FMD), concomitant extra-coronary FMD was reported in 72.3% of patients with SCAD, other extra coronary vascular abnormalities (EVA) were also reported as potential association with SCAD. It is possible that these lesions play a role in potentiating the dissection, or increasing the tortuosity reported in the coronary vessels [3]. SCAD has also been associated with pregnancy and in the peripartum period, as 27%-43% of patients with acute myocardial infarction (MI) during pregnancy has SCAD, and it comprises about 5%-17% of all SCAD patients, likely due to the effect of pregnancy hormones on the connective tissues [4]. Mutations in single genes like F11R, PHACTR1, and TSR1 were all linked to the development of SCAD [5]. Other connective tissue diseases like Marfan and Ehler-Danlos syndrome were also considered as potential risk factors. There are also some rare reported cases that suggest an association with hyperhomocysteinemia, high-intensity exercise, substance abuse especially amphetamine, systemic inflammatory conditions, severe acute respiratory syndrome coronavirus 2 (SARS-COV-2) infection, and hormone replacement therapy. Our patient did not have a medical history of any of the risk factors, although we did not screen her for the presence of FMD in other blood vessels such as the renal and cerebral arteries. 

The clinical presentation of SCAD is similar to the presentation of ACS, as patients may present as chest pain with EKG changes, cardiogenic shock, ventricular arrhythmias with or without cardiac arrest, and heart failure. Our patient’s presentation was unusual, as she presented with nonspecific chest pain and syncope with negative initial cardiopulmonary workup, including EKG and cardiac enzymes to rule out ACS. The following day, she developed sudden cardiac arrest. This in fact is diagnostically challenging, as physicians tend to discharge many young patients with chest pain and negative cardiac workup with reassurance.

Most patients with SCAD are treated conservatively with either dual or single antiplatelet therapy and anticoagulation. Beta-blockers were shown to be protective against recurrence of SCAD [6]. Percutaneous coronary intervention (PCI) and coronary artery bypass graft (CABG) are other options of treatment. The rate of complications of PCI in SCAD is high compared to PCI in atherosclerosis, hence the trend to treat patients conservatively [7]. However, in the presence of hemodynamic compromise, critical luminal obstruction, and proximal dissections, PCI may be reasonable. The use of IABP is also controversial; some studies reported worsening of the dissection with the use of IABP while others reported IABP to be safely used with good outcomes [8]. It has been reported that 13% of SCAD cases had left main dissection and almost all these patients usually present with cardiogenic shock [9]. 

## Conclusions

Spontaneous coronary artery dissection is an uncommon cause of acute coronary events. It should be suspected especially among young middle-aged females with potential risk factors although it may not always be associated with any risk factors like in our case. The clinical presentations are different which require a very high index of suspicion for timely diagnosis. There are no well-established standardized treatment guidelines to satisfy each clinical presentation. Timely diagnosis and appropriate treatment based on the severity of presentation have resulted in favorable outcomes. 

## References

[1] Jackson R, Al-Hussaini A, Joseph S (2019). Spontaneous coronary artery dissection: pathophysiological insights from optical coherence tomography. JACC: Cardiovascular Imaging.

[2] Hayes SN, Tweet MS, Adlam D, Kim ES, Gulati R, Price JE, Rose CH (2020). Spontaneous coronary artery dissection: JACC state-of-the-art review. J Am Coll Cardiol.

[3] Haque N, Harzand A, Kim E (2020). The presence of extra coronary vascular abnormalities is associated with spontaneous coronary artey dissection recurrence. J Am Coll Cardiol.

[4] Nallapati C, Park K (2021). Ischemic heart disease in pregnancy. Cardiol Clin.

[5] Sun Y, Chen Y, Li Y (2019). Association of TSR1 variants and spontaneous coronary artery dissection. J Am Coll Cardiol.

[6] Saw J, Starovoytov A, Humphries K (2019). Canadian spontaneous coronary artery dissection cohort study: in-hospital and 30-day outcomes. Eur Heart J.

[7] Kotecha D, Garcia-Guimaraes M, Premawardhana D (2021). Risks and benefits of percutaneous coronary intervention in spontaneous coronary artery dissection. Heart.

[8] Matsuo Y, Ozaki K, Ikegami R (2021). Conservative treatment with an intra-aortic balloon pump to treat acute myocardial infarction due to spontaneous coronary artery dissection. J Cardiol Cases.

[9] Lobo AS, Cantu SM, Sharkey SW (2019). Revascularization in patients with spontaneous coronary artery dissection and ST-segment elevation myocardial infarction. J Am Coll Cardiol.

